# Nuclear factor erythroid 2 – related factor 2 and its relationship with cellular response in nickel exposure: a systems biology analysis

**DOI:** 10.1186/s40360-019-0360-4

**Published:** 2019-12-19

**Authors:** Luisa Jiménez-Vidal, Pedro Espitia-Pérez, José Torres-Ávila, Dina Ricardo-Caldera, Shirley Salcedo-Arteaga, Claudia Galeano-Páez, Karina Pastor-Sierra, Lyda Espitia-Pérez

**Affiliations:** 1 Facultad de Ciencias de la Salud, Grupo de Investigación Biomédica y Biología Molecular, Universidad del Sinú, Calle 38 Cra 1W, Barrio Juan XXIII, Montería, Córdoba Colombia; 2 Unit for Development and Innovation in Genetics and Molecular Biology, Universidad Simón Bolívar, Barranquilla, Atlántico, Colombia; 3 Facultad de Ciencias de la Salud, Grupo de Investigación en Enfermedades Tropicales y Resistencia Bacteriana, Universidad del Sinú, Montería, Córdoba Colombia

**Keywords:** Nickel, Nrf2, ER-stress, UPR, NiSO_4_, Ni_3_S_2_

## Abstract

**Background:**

Nickel and nickel-containing compounds (NCC) are known human carcinogens. However, the precise molecular mechanisms of nickel-induced malignant transformation remain unknown. Proposed mechanisms suggest that nickel and NCC may participate in the dual activation/inactivation of enzymatic pathways involved in cell defenses against oxidative damage, where Nuclear factor-erythroid 2 related factor 2 (Nrf2) plays a central role.

**Methods:**

For assessing the potential role of proteins involved in the Nrf2-mediated response to nickel and NCC exposure, we designed an interactome network using the STITCH search engine version 5.0 and the STRING software 10.0. The major NCC-protein interactome (NCPI) generated was analyzed using the MCODE plugin, version 1.5.1 for the detection of interaction modules or subnetworks. Main centralities of the NCPI were determined with the CentiScape 2.2 plugin of Cytoscape 3.4.0 and main biological processes associated with each cluster were assessed using the BiNGO plugin of Cytoscape 3.4.0.

**Results:**

Water-soluble NiSO_4_ and insoluble Ni_3_S_2_ were the most connected to proteins involved in the NCPI network. Nfr2 was detected as one of the most relevant proteins in the network, participating in several multifunctional protein complexes in clusters 1, 2, 3 and 5. Ontological analysis of cluster 3 revealed several processes related to unfolded protein response (UPR) and response to endoplasmic reticulum (ER) stress.

**Conclusions:**

Cellular response to NCC exposure was very comparable, particularly concerning oxidative stress response, inflammation, cell cycle/proliferation, and apoptosis. In this cellular response, Nfr2 was highly centralized and participated in several multifunctional protein complexes, including several related to ER-stress. These results add evidence on the possible Ni^2+^ induced – ER stress mainly associated with insoluble NCC. In this scenario, we also show how protein degradation mediated by ubiquitination seems to play key roles in cellular responses to Ni.

## Backgrounds

Nickel-containing compounds (NCC) are widely used in numerous industrial process while metallic nickel is a known human carcinogen [1]. However, the precise molecular mechanisms of nickel-induced malignant transformation remain unclear [2]. Toxicological profiles of NCC depend on their soluble and insoluble characteristics, which in turn affect its cellular uptake, and therefore the fate of dissolved nickel ion Ni^2+^ (carcinogen) [3]. Insoluble NCCs (i.e., Ni_3_S_2_, Ni (CO)_4_) are taken up inside the cell via phagocytosis, were slow endosome solubilization leads to Ni^2+^ dissociation and aggregation near the nucleus, directly interacting with DNA [4].

In contrast, soluble compounds (i.e., NiSO_4_, NiCl_2_) are incorporated via calcium/magnesium-dependent membrane channels, or the proton-coupled divalent cation transporter (DMT-1) and therefore affecting cytoplasmic proteins, which eventually causes increase cytotoxicity [5]. Cellular events following nickel and NCC exposure can be categorized as disrupted cell proliferation, metabolic modifications, oxidative stress, inflammation and cell death [6]. NCC can affect cellular iron uptake activating hypoxia signaling pathways (directly related nickel-induced carcinogenesis) and induce chemokine and cytokine gene expression (nickel-driven correlated pathway to immune response) [7, 8]. Several studies have concluded that production of reactive oxygen species (ROS) may also contribute to nickel-induced carcinogenesis [7], and recent studies have shown a link between environmental exposure to nickel compounds and increased oxidative DNA damage [9]. Proposed mechanisms suggest that nickel and nickel compounds may participate in the dual activation/inactivation of enzymatic pathways involved in cell defenses against oxidative damage, where the Nuclear factor-erythroid 2 related factor 2 (Nrf2) plays a central role [10]. Other reports indicate that Ni^2+^ at sublethal concentrations of 10–50 μM resembling in vivo tissue deposition can activate the Nrf2 signaling pathway in human monocytic cells by increasing whole-cell Nrf2 levels and nuclear translocation [11]. Nrf2 is a transcription factor that upregulates antioxidant response elements (AREs)-mediated expression of antioxidant enzyme and cytoprotective proteins, which protects the cells against oxidative injury in tissues and ROS [12]. Nrf2 has been traditionally considered as a tumor suppressor; however, several recent studies demonstrate that high Nrf2 overexpression may protect cancer cells against oxidative stress and chemotherapeutic agents [13]. Under oxidative stress conditions, Nrf2 is translocated to the nucleus, where it binds to AREs in the promoter regions of genes encoding antioxidant proteins and detoxification enzymes to initiate their transcriptions [14]. Additionally, recent evidence supports that nickel-induced Nrf2 expression inhibits apoptosis and promotes autophagy, which may explain nickel tumorigenesis in vitro [15].

The use of hierarchical clustering analysis alongside with knowledge-based pathway information provides a comprehensive insight into the molecular mechanisms underlying metal-induced toxicity [16]. Thus, the present study aimed to evaluate the interaction between NCC, and proteins associated with the transcription factor Nrf2 using a systems biology approach, to elucidate how nickel and NCC exposure can affect the antioxidant response of the cell. The understanding of the complexity of the Nrf2 interactome could improve the identification of its role in the nickel-induced appearance of diseases such as cancer.

## Methods

### Construction of the chemo-biology network between nickel compounds and Nrf2-mediated response proteins

For assessing the potential role of proteins involved in the Nrf2-mediated response to nickel and NCC exposure, we designed an interactome network using the STITCH search engine version 5.0 and the STRING software 10.0 [17, 18]. While STITCH allows visualization of the physical interactions between the chemical elements and the proteins, the STRING software generates the protein-protein interactions [19].

Main NCC with potential toxicological characteristics detected in the workplace of the nickel-mining areas or nickel-using industries and its surrounding environments, [20–22] were used for network prospecting in STITCH. Compounds that were not present in the STITCH database (or those that did not show any protein binding) were excluded from the analysis. The parameters used to prospect the networks for *Homo sapiens* in STITCH software were: no more than 50 interactions; high confidence score (0.700); and network depth equal to 2; all active prediction methods enabled except text mining. Chemical names, CAS, synonyms and molecular formulae for the resulted NCC are presented in Table 1.

**Table 1:** Chemical names and CAS names, synonyms, solubility and molecular formulae of selected nickel compounds

Chemical name	CAS Reg. No^a^	Synonyms	Formula	WSTC^b^ (mg/L)
Nickel subsulfide	12035–72-2	Nickel sesquisulfide; tri-nickel disulfide; nickel subsulfide (Ni_3_S_2_); nickel sulfide (Ni_3_S_2_)	Ni_3_S_2_	517 (37 °C) (Water-insoluble)
Nickel chloride	7718-54-9	Nickel (II) chloride; nickel (2+) chloride; nickel chloride (NiCl_2_); nickel dichloride; nickel dichloride (NiCl_2_); nickelous cholride	NiCl_2_	2,5E6 (20 °C) (Water-soluble)
Nickel sulfate	7786-81-4	Nickel monosulfate; nickelous sulfate; nickel sulfate (1:1); nickel (II) sulfate; nickel (2+) sulfate; nickel (2+) sulfate (1:1); nickel sulfate (NiSO_4_); sulfuric acid, nickel (2+) salt (1:1)	NiSO_4_	2,9E5 (20 °C) (Water-soluble)
Nickel carbonyl	13463–39-3	Nickel carbonyl (Ni(CO)_4_); nickel tetracarbonyl; tetracarbonylnickel; tetracarbonylnickel (0)	Ni (CO)_4_	20 (20 °C) (Water-insoluble)

^a^ [21]

^b^ Water Solubility Temperature Corrected

Protein interactions for Nrf2 were predicted in STRING 10.0. The parameters used to prospect the networks for *Homo sapiens* in STRING software were: no more than 50 interactions; high confidence score (0.700); and network depth equal to 2; all active prediction methods enabled except text mining. To identify additional interactors for Nrf2, we downloaded additional Nrf2 interactions deposited in Bio-GRID [23]. Each network generated by STITCH and STRING was combined in a single major NCC-protein interactome (NCPI) using the advanced network merge function of the Cytoscape 3.6.1 software [24].

### Modular analysis of the interactome network

The major NCPI generated was analyzed using the MCODE (Molecular Complex Detection) plugin, version 1.5.1 for the detection of interaction modules or subnetworks (i.e., intensively connected parts of the network) [25]. Interaction modules may represent molecular complexes [26]. As previously described [27], t loops included; cutoff degree 2; deletion of single connected nodes from cluster (haircut option enabled); expansion of cluster by one neighbor shell allowed (fluff option enabled); node density cutoff 0.1; node score cutoff 0.2; kcore 2; and maximum depth of network 100. Compounds that were not present in the STITCH database (or those that did not show any protein binding) were excluded from the analysis.

### Centralities analysis

To identify which nodes have a central position within the network, we performed the calculation of the main centralities of the network (degree, closeness and betweenness) with the CentiScape 2.2 plugin of Cytoscape 3.0 [28]. Betweenness indicates the number of the shortest paths that go through each node. Nodes with high betweenness scores are responsible for controlling the flow of information through the network topology; thus, more information will pass through that node [19]. Nodes with a high betweenness score are called bottleneck nodes (BN) and can represent essential proteins in signaling pathways [29]. On the other hand, node degree is a measure that indicates the number of connections associated with a specific node, which in turns, is also equal to the number of neighbors of the node [30]. Nodes with a relatively higher degree are termed hubs (H) [28] and have critical regulatory functions in the cell [19]. Consequently, a node with hub-bottleneck (H-BN) characteristics is considered a key regulator of a particular biological process and essential for the flow of information through the network [31].

### Gene ontology (GO) analysis

In addition to the MCODE analysis, we analyzed the biological processes [32] associated with each cluster using the Biological Network Gene Ontology (BiNGO) plugin of Cytoscape 3.4.0 [33]. The degree of functional enrichment for a given group and category was assessed quantitatively (*p*-value) using a hypergeometric distribution. The correction of the multiple tests was also evaluated by applying the false discovery rate (FDR) algorithm [34], which was fully implemented in the BiNGO software at a significance level of *p* < 0.05.

## Results

**Fig. 1:**
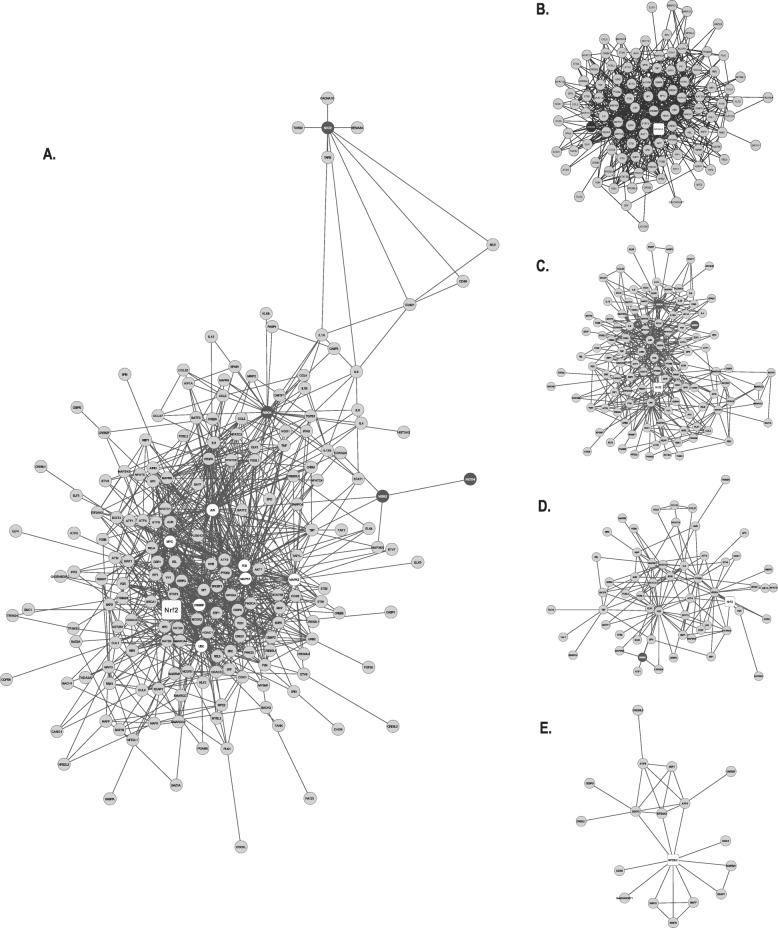
The protein-protein interacting network of Ni_3_S_2_, NiCO_4_, NiSO_4,_ and NiCl_2_ with *Homo sapiens* proteins involved in the Nrf2-mediated response to nickel exposure. **a** The main network showing the NCC (black nodes) with 188 nodes and 915 edges (connections). HBs are represented by white nodes. Nrf2 appears as a round rectangle in white. **b** Cluster 1 composed of 114 nodes; 732 edges and associated to NiSO_4_. **c** Cluster 2 composed of 113 nodes; 387 edges and associated to NiSO_4_ and Ni_3_S_2_. **d** Cluster 3 composed of 55 nodes; 129 edges and associated to Ni_3_S_2_. **e** Cluster 5 composed of 18 nodes; 29 edges and no association with any NCC

The major NCPI generated for potential role proteins involved in the Nrf2-mediated response to NCC exposure included 188 nodes and 915 edges (Fig. 1). Water-soluble NiSO_4_ and insoluble Ni_3_S_2_ were observed as the most protein linked NCC involved in the NCPI network. NiSO_4_ is connected to 32 proteins with 118 connections and Ni_3_S_2_ is connected to 6 proteins with 11 connections.

**Fig. 2:**
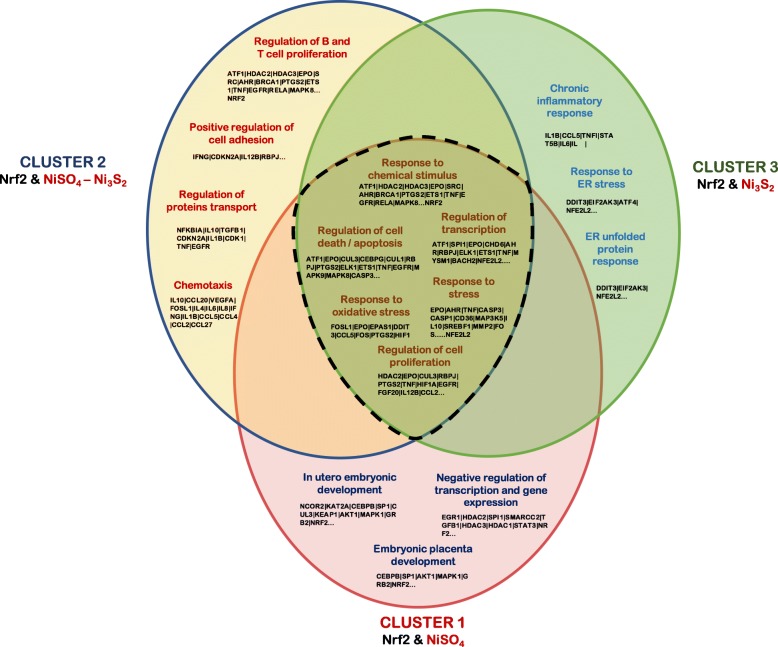
Venn diagram for common and non-overlapping (unique) annotations of clusters 1, 2 and 3. Dashed section include common annotations to all clusters. Beside the annotations found in the dashed section, no other common annotations were found between clusters 1–3 and 2–3 (0)

The use of MCODE established the presence of 5 protein modules with a coefficient of cohesion greater than or equal to 3.0. Our analyses showed that in the cellular response to nickel exposure, Nfr2 is highly central and participates of several multifunctional protein complexes, as demonstrated by its simultaneous presence in clusters 1, 2, 3 and 5 (Fig. 2). It should be noted that after the MCODE analysis other NCC used in the initial prospection such as NiCO_4_ did not display any clusterization, while NiCl_2_ was found clustered in module 6 below the cutoff score and were excluded from the analysis. Additionally, cluster 5 did not show association to any NCC; thus, ontological processes related to this cluster were used only to discuss its relationship with cluster 3.

The system chemo-biology analysis of clusters 1, 2 and 3 revealed several common annotations: *(i) regulation of transcription, (ii) response to stress, (iii) regulation of cell proliferation, (iv) response to chemical stimulus, (v) regulation of cell death and (vi) response to oxidative stress.* All biological process linked to each of these clusters and its respective proteins are presented in (Additional file 1). In cluster 1, Nrf2 appeared associated with NiSO_4_, while in cluster 2 was connected to Ni_3_S_2_ and NiSO_4_, and in cluster 3 only to Ni_3_S_2_.

Beside the common annotations described for all the clusters, we were interested in identifying mechanisms of toxicity that were unique to each NCC. Thus, a more detailed analysis of the GO terms in each cluster looking for non – overlapping (unique) annotations, revealed that in cluster 1, NiSO_4_ differentially clustered in association with proteins involved with the negative regulation of transcription and gene expression, and interestingly, to proteins related to the development of the fetus and placenta (Fig.2). In this cluster, first Nrf2 neighboring proteins were also associated with the regulation of transcription and translation and response to stress (Data not shown).

In cluster 3, Ni_3_S_2_ were observed to be connected distinctly with proteins associated with the chronic inflammatory response and the response to ER stress, including the unfolded protein response. In cluster 2, the presence of both NCC seems to cause a distinct clusterization around proteins associated with the immune and inflammatory response (Fig. 2). Additional biological process linked to cluster 3 and its respective proteins are presented in (Additional file 1: Table S3). In this particular cluster, Nrf2 appears as a connection protein between nodes involved in stress response and detoxification pathways (MAFG, MAFK, MAFF) and activation of signaling proteins involved in unfolded protein and DNA damage response (XBP1, ATF4, DDIT3, EIF2AK3).

**Fig. 3:**
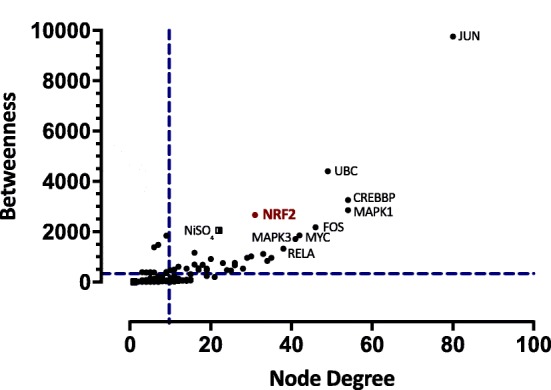
Analysis of centrality of proteins and selected Ni compounds in the interaction network_._ Blue dashed lines represent the threshold value for node degree and betweenness. Circular dots represent proteins, Ni compounds are indicated by square dots

**Fig. 4:**
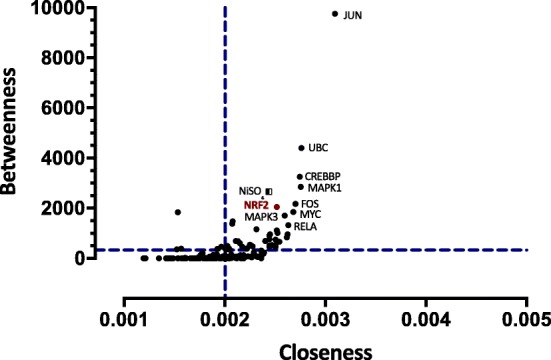
Analysis of centrality showing node degree and betweenness relationship of proteins and selected Ni compounds. Blue dashed lines represent the threshold value. Circular dots represent proteins, Ni compounds are indicated by square dots

**Fig. 5:**
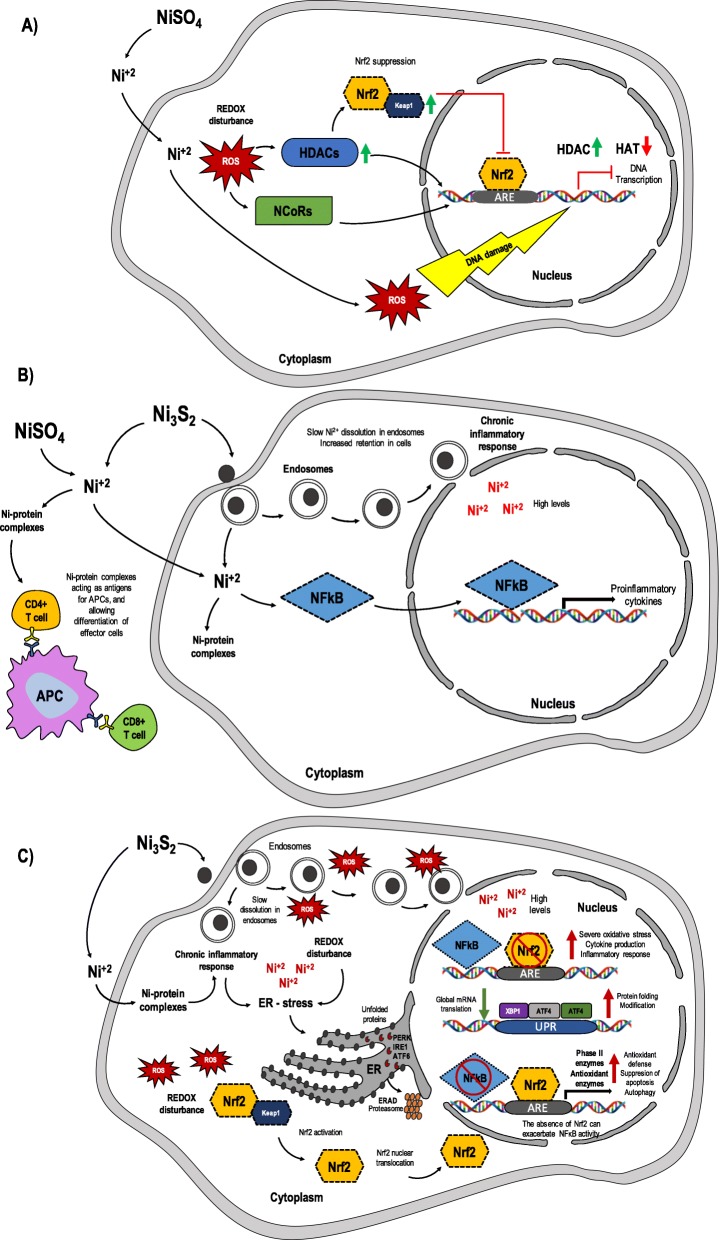
Molecular models of the potential role of proteins involved in the Nrf2 – mediated response to NCC exposure. **a** NiSO_4_ allows the release of Ni^2+^ ion which increases ROS production in the cytoplasm, inducing 1) hypomethylation and HAT inhibition through increased HDACs and NCoRs activity, which reduces Keap1/Nrf2 dissociation and posterior Nrf2/ARE transcription, and 2) inhibition of DNA repair process; **b**) Ni_3_S_2_ uptake is facilitated via phagocytosis, delaying Ni^2+^ dissolution inside the cytoplasm. However both Ni_3_S_2_ and NiSO_4_ releases Ni^2+^ outside alveolar cells increasing production of Ni-protein complexes which acts as antigens for APC cells following differentiation to Ni^2+^-specific interferon γ (IFN-γ)-producing effector T cells, whereas inside the cell increase NF-kB transcription and proinflammatory response; **c**) Ni_3_S_2_ is capable of the release of high levels of the Ni^2+^ ion and ROS as a product of Ni_3_S_2_ mediated-phagocytosis. Retention of Ni_3_S_2_ and slow dissolution in endosomes are the primary cause of a possible chronic inflammation response. Ni^2+^ from Ni_3_S_2_ can induce ER stress through the activation of PERK, IRE1, and ATF6 pathways. During ER-stress conditions proteins that fail the ER quality control are transported back to the cytosol where they are rapidly destroyed by the ER-associated degradation (ERAD) pathway. Additionally, redox status generated by Ni^2+^ regulates the nuclear activity of NF-kB proteins by altering the binding of NF-kB proteins to DNA in part via the Nrf2-signaling pathway. The absence of Nrf2 can exacerbate NF-κB activity leading to increased cytokine production

To identify the significant nodes within the NCPI network, we calculated node degree, closeness, and betweenness centralities. From these analyses, two graphs were obtained containing the proteins that showed the highest centrality values (Fig.3 and Fig.4). From these analyses, we identified 10 major nodes with the highest centrality values and H-BN characteristics: JUN, UBC, CREBBP, MAPK1, Nrf2, FOS, NiSO_4_, MYC, MAPK3 and RELA (Fig. 3). Proteins described in clusters 1, 2, 3 and 5 and those with the highest centrality values were used in the design of a molecular model for potential role proteins involved in the Nrf2-mediated response to NCC exposure (Fig. 5).

## Discussion

Considering that toxicological profiles of water soluble and insoluble NCC differ substantially, we decided to include both kinds of compounds in our analysis. However, only NiSO_4_ and Ni_3_S_2_ were observed as the most connected to proteins involved in the NCPI network. NiSO_4_ is a highly water-soluble nickel salt that causes a rapid release of Ni (II) ions after inhalation, while Ni_3_S_2_ is poorly soluble in water [3]. Soluble nickel particles can be dissolved in the lung and intestine tissues, and the nickel ions are quickly removed by ciliary transport. In contrast, less soluble nickel particles can enter epithelial cells by phagocytosis [35] where they are gradually dissolved, providing a constant source of nickel ions [36]. According to this “Ni ion bioavailability”–model, the carcinogenic potential of Ni depends on the availability of Ni ions in the cell nucleus [37]. Thus, NCC with the greatest potential to induce tumors would be those insoluble enough to be present in tissues as particles incorporated into epithelial cells via phagocytosis, and once inside the phagosomes capable of a slow, yet sustainable release over time of the Ni^2+^ ion [38, 39]. Phagocytic cells such as neutrophils and macrophages are important sources of endogenous oxygen radicals [40]. Thus, phagocytized nickel species, such as Ni_3_S_2_ and NiS produce more oxidative stress due to particle-induced irritation inside the cell [41, 42]; nevertheless, other NCC like NiSO_4_ are also capable of inducing oxidative stress, especially in the presence of H_2_O_2_ [43]. Under these oxidative stress conditions, Nrf2 translocation to the nucleus activates ARE-mediated transcriptional response [14].

In our system chemo-biology analysis, Nrf2 appears as highly central protein, participating simultaneously of several multifunctional protein complexes. Remarkably, those cellular responses attributed to NiSO_4_ and Ni_3_S_2_ were very similar among them particularly concerning oxidative stress response, and cell cycle/proliferation. However, a more detailed analysis of the GO terms in each cluster looking for non – overlapping (unique) annotations, revealed that in cluster 1, Nrf2 and NiSO_4_ differentially clustered with proteins related to the development of the fetus and placenta. Regarding developmental toxicity, recent evidence supports that soluble Ni^2+^ can affect organogenesis in vivo [44–46], although nowadays no substantial epidemiological evidence has demonstrated a causal link between NiSO_4_ and developmental effects [9]. As a contact allergen, NiSO_4_ poorly correlated with Keap1/Nrf2 expression during dendritic cell activation in vitro [47]. However, nickel is capable of inducing lipid peroxidative damage in human placenta membrane, probably related to decreased placental viability, altered permeability and potentially subsequent embryotoxicity [48]. Previous studies demonstrated that nickel could decrease both intracellular glutathione levels and enzymatic activity of superoxide dismutase, glutathione peroxidase and catalase, which leads to ROS production and an increase in intracellular oxidative stress [49]. In this scenario, Nrf2 constitutes an essential regulator of placental development and growth. In a recent study, Kweider et al., [50] investigated the effects of Nrf2 on fetal-placental development in pregnant Nrf2 knockout and Nrf2 wild-type mice, finding that a deficiency in Nrf2 signaling can increase the level of oxidative stress and may negatively affect nutrient transfer capacity, affecting fetal growth demands. Other reports have suggested that nickel ions affect fetal development by altering maternal endocrine status leading to hyperglycemia [51]. As most of these results in human placenta have been reported for Ni and NiCl_2_, our analysis would suggest similar effects for NiSO_4_, which may suggest a potential new role of Nrf2-NiSO_4_ correlation in other tissues during developmental stages.

In the same cluster 1, Nrf2 and NiSO_4_ also differentially clustered in association with proteins involved with the negative regulation of transcription and gene expression. Several studies have also demonstrated that nickel species are also capable of negatively regulate transcription and gene expression through epigenetic mechanisms [52]. This aberrant histone modifications have been found to be associated with various human diseases, including cancer [53] and is considered as one of the possible mechanisms behind the potent carcinogenic effects of NCC [54]. Previous studies have shown that cellular exposure to NCC causes the intracellular accumulation of Ni ions and a loss of acetylation at all four core histones with global decreased DNA methylation [55, 56]. The evidence demonstrated that NCC exposure decreases histone acetylation by inhibiting histone acetyltransferase (HAT) activity but has no effects on histone deacetylases (HDAC) [57]. In this sense, within cluster 1 several class I HDAC (HDAC1, HDAC2, HDAC3) were identified, together with proteins with histone deacetylase activity like NCoR-1 and NCoR-2. Nickel species induce histone hypoacetylation through a multi-pathway, where cellular exposure to Ni^2+^ inhibits HAT activity via ROS production [57]. In this scenario, in vitro studies in cell lines of airway epithelial cells, demonstrated that increase HDAC activity correlated with lower levels of Nrf2 in parallel with elevated Nrf2 acetylation [58]. In line with this, other reports found that HDAC inhibition reduced the expression of Keap1, inducing Keap1/Nrf2 dissociation, Nrf2 nuclear translocation, and Nrf2 binding to ARE [59]. Thus, we can suppose that cellular exposure to NiSO_4_ could increase HDAC activity and downregulation of the Nrf2 levels, leading to oxidative damage, lipid peroxidation and DNA damage [60]. These modifications may at least partially account for NiSO_4_ carcinogenic activities. Another possible explanation for NiSO_4_ negative regulation of the transcription process in cluster 1, could include the direct effect of NiSO_4_ on intracellular ROS generation and DNA damage repair inhibition [43]. In response to various types of DNA lesions, local inhibition of transcription may occur to prevent the production of aberrant transcripts and to avoid interference between transcription and repair machinery [61]. Additionally, considering that chromatin accessibility occurring after histone acetylation would allow repair proteins to gain access to the damaged lesion [62], the inhibition of HAT together with the increased activity of the HDAC may difficult the access of repair machinery to the damage inhibiting the repair process. In this regard, NCC and particularly NiSO_4_ have been consistently associated with a decreased DNA repair capacity [43] that also can lead to a reduced transcription rate while the cell repairs the damaged DNA.

In cluster 2, Nrf2, NiSO_4_, and Ni_3_S_2_ clustered in association with proteins involved with the immune and inflammatory response (TNF, NF-kB, IL4, IL6, IL8, and IFN) typically related with hypersensitivity to Ni-species. Both NiSO_4_ and Ni_3_S_2_ are potent metallic allergens [53]. In NiSO_4_ and other soluble NCC, Ni^2+^ can bond to soluble proteins on antigen-presenting cells (APC) [63]. APCs recognize this Ni^2+^-protein complex as an antigen, causing the activation of naïve T cells [64], which subsequently differentiate to Ni^2+^-specific interferon γ (IFN-γ)-producing CD4+ and CD8+ effector T cells [65], involved in Ni allergies. Ni^2+^ also activates the expression of intercellular adhesion molecules on endothelial cells and keratinocytes, therefore amplifying the inflammatory response [66]. More recently, Ni^2+^ and particularly NiSO_4_ has been shown to alter inflammatory cytokine secretion from monocytes [67] by modulating transcription factor signaling pathways such as the nuclear factor kappa B (NF-kB), that regulate cytokine secretion [68, 69]. However, Ni^2+^ does not alter the activation or nuclear translocation efficiency of NF-kB [70]; emerging evidence suggest that redox status generated by Ni^2+^ regulates the nuclear activity of NF-kB proteins [71] by altering the binding of NF-kB proteins to DNA [70]. These reports suggest that Ni^2+^ could exert its effects on cytokine secretion at least in part via the Nrf2-signaling pathway [11, 71]. Furthermore, although Nrf2 absence can exacerbate NF-κB activity by increasing cytokine production [72, 73], NF-κB can modulate Nrf2 transcription and activity, having both upregulation and downregulation effects on the target gene expression [74].

In cluster 3, Ni_3_S_2_ were observed to be connected distinctly with proteins associated with the chronic inflammatory response. In contrast to soluble NCC, Ni_3_S_2_ could be retained in lung cells for a sufficient time to exacerbate the development of inflammation response [75] and activate various signaling pathways that enhance allergic reactions as the second signal [76]. A chronic inflammation process is in accordance with the water-insolubility properties of Ni_3_S_2_ discussed above, which explains why inhalation of this form of nickel might be more related with a chronic response than inhalation of soluble nickel compounds [77].

Ni_3_S_2_ were also related to endoplasmic reticulum (ER) stress response, including the unfolded protein response (UPR). Several of these processes related to the response to ER stress were also found in cluster 5, although without association with any NCC. ER is the site of intracellular calcium regulation, synthesis, and folding of secretory proteins, which is crucial for cellular homeostasis [78]. Many disturbances, including those of cellular redox regulation, cause accumulation of unfolded proteins in the ER, affecting a variety of cellular signaling processes, including redox homeostasis, energy production, inflammation, differentiation, and apoptosis [79]. In consequence, ER stress activates the UPR to reestablish normal ER function by enhancing ER protein folding, inhibiting global mRNA translation, and reducing the influx of new proteins into the ER, which disposes terminally misfolded proteins by ER-associated protein degradation (ERAD) and autophagy [78]. Excessive and prolonged ER stress triggers cell suicide, usually in the form of apoptosis.

Although recent reports have demonstrated that As, Cd, Ag, Mn, and Cu can induce ER stress [80–83], there is limited evidence on Ni and NCC induced – ER stress, the latter only confirmed for nickel acetate in vitro [84] and NiCl_2_ in vivo [85]. Based on our results and considering that metal ions can cause protein misfolding and aggregation by *i)* inhibiting the refolding of chemically denatured proteins in vitro*, ii)* interfering with protein folding in vivo and *iii)* causing the aggregation of nascent proteins in living cells [86], we can hypothesize that Ni^2+^ can activate the response to ER stress by meanings of any of these mechanisms. Mainly, Ni_3_S_2_ has been associated with a more efficient uptake and delivery of Ni^2+^ ion to the cell nucleus in vitro compared to soluble NCC like NiSO_4_ [87]. Additionally, Ni_3_S_2_ can indirectly induce ROS via inflammation and phagocyte activation, whereas directly through H_2_O_2_ formation [88]. Accumulating evidence suggests that protein folding and generation of ROS as a byproduct of protein oxidation in the ER are closely linked events [89]. It has also become apparent that UPR activation mediated by oxidative stress is an adaptive mechanism to preserve cell function and survival. In fact, persistent oxidative stress and subsequent ER stress and Nrf2 activation, initiate apoptotic cascades and are now known to play predominant roles in the pathogenesis of multiple human diseases including cancer, diabetes, atherosclerosis, and neurodegenerative diseases [90, 91]. However, the particular capacity of Ni_3_S_2_ induced ER stress and UPR response is of particular interest and needs to be further evaluated by in vitro studies.

Finally, to identify the major nodes within the NCPI, we calculated node degree, closeness and betweenness centralities. From these analyses, two graphs were obtained containing the proteins that showed the highest centrality values (Fig.3 and Fig.4). Interestingly, the same nodes showed similar relevance in both graphics. Thus, the joint analysis allows us to establish proteins that make up the most relevant network (hub-bottlenecks - HB). We identified several JUN, UBC, CREBBP, MAPK1, Nrf2, FOS, MYC, RELA, MAPK3 and NiSO_4_ as HB. JUN (c-Jun, JunB, and JunD) and FOS family members (c-Fos, FosB, Fra1 and Fra2) constitute a mixture of homo and heterodimers that composed the AP-1 transcription factor. The AP-1 transcription factor participates in the control of cellular responses to stimuli that regulate proliferation, differentiation, immune response, cell death and the response to genotoxic agents or stress, all related with NCC response according to our results [92]. Apart from dimerize between each other, JUN and FOS proteins also dimerize efficiently with other transcription factors such as members of the ATF/CREB [93]. In fact, AP-1 can be induced in response to certain metals including nickel [94]. Expression of JunB was significantly up-regulated in some nickel exposures [95]. In human airway epithelial cells (BEAS-2B), c-Jun and c-Fos mRNA levels were induced after the addition of nickel [96]. AP-1 is a redox-sensitive transcription factor, both ROS-dependent and ROS-independent pathways may play a role in activation of AP-1 in nickel-exposed cells [97, 98]. This ROS-mediated activation of AP-1 is modulated by the transcription activation of APE, the redox regulator of AP-1 and is mediated via activation of c-Jun/ATF-2 that binds to the CREB binding site within the human APE promoter [99]. CREB is a leucine zipper-type transcription factor also found as HB in the NCPI, ubiquitously expressed in organs [100]. CREB has many functions in cells, including regulation of cell growth, differentiation and stress responses in various cell types, and recently has been strictly associated with the ER stress [101]. In line with this, another HB protein, ubiquitin C (UBC) has been shown to be involved in the regulation of various biological processes related to nickel-induced toxicity [102], and other biological processes such as the cell cycle, apoptosis, and inflammation, which are contributors to human cell toxicity and carcinogenicity [103]. The ubiquitin – proteasome pathway (UPP) catalyzes the selective degradation of short-lived proteins and the elimination of proteins with abnormal conformation [104], including those that accumulate in ER during ER-stress conditions; this process is known as ER-associated degradation (ERAD), proteins that fail the ER quality control are transported back to the cytosol where they are rapidly destroyed by the UPP [105]. MYC is a family of proto-oncogenes that code for transcription factors, known to induce both increase proliferation and apoptosis depending on cellular status [106]. Particularly, c-Myc functions as a transcription factor and induces proliferation, energy metabolism [107], and ribosome biogenesis [108]. This ribosome biogenesis has been associated with an enhanced protein synthesis, required for MYC oncogenic activity [109, 110] and more recently with UPR activation [106]. Thus, UPR would act as an enhancer of c-Myc–induced transformation, suggesting that UPR inhibition may be particularly effective against malignancies characterized by c-Myc overexpression [106]. RELA is one of the five different proteins of the NF-kB factor previously discussed, also called p65 subunit. p65 plays a critical role in inducing target genes of NF-kB; recent evidence has demonstrated the translocation of NF-κB p65 following ER stress [111]. Additionally, this subunit has been shown to function as a negative regulator of Nrf2 activation either by depriving CREB binding protein from Nrf2 or by recruitment of histone deacetylase 3 (HDAC3), a corepressor to ARE [112]. In another study it was shown that p65 decreased NRF2 through the interaction of Keap1, inhibiting Nrf2-ARE pathway [74].

## Conclusions

Our analysis based on system chemobiology tools suggest that during NCC exposure, Nfr2 would play a pivotal role during cellular transcriptional, regulatory and chemical responses, processes correlated with cell proliferation, death, and oxidative stress. Our results add some evidence to soluble NCC and its relationship with modification of the epigenetic response regarding ROS-mediated histone acetylation/deacetylation, which may be related to Nrf2 suppression, a process that may be surpassing DNA repair mechanisms inside the cell. Compared with the response to water-soluble NCC exposure, Nrf2 seems to play a more significant role in response to insoluble NCC where phagocytized nickel species may produce and increase oxidative stress, chronic inflammation and ER- stress. These results may suggest a model where Ni exposure may cause persistent oxidative stress, subsequent ER stress, and Nrf2 activation, adding evidence on the possible Ni^2+^ induced – ER stress. Additionally, in this model, protein degradation mediated by ubiquitination seems to play key roles in cellular responses to Ni. Thus, our results would suggest that water-insolubility of NCC plays a major role in predominant cell damage regarding ER stress, and in general, a systemic effect of NCC based on solubility as revealed by cluster analysis.

Furthermore, our model provides new insights regarding how NCC obtained as by-products in many industrial processes may cause affectations regarding chronic response to several diseases.

## Supplementary information

**Additional file .** clusters ontology analysis.

## Data Availability

The databases constructed and used in this article are public and available in the supplemental material published.

## Abbreviations

ARE: antioxidant response elements
ER: endoplasmic reticulum
HAT: histone acetyltransferase
HDAC: histone deacetylase
NCC: nickel containing compounds
NCPI: NCC-protein interactome
NiSO_4_: nickel sulfate; Ni_3_S_2_ nickel subsulfate; NiCl_2_: nickel chloride; Ni (CO)_4_: nickel carbonyl.
Nrf2: Nuclear factor-erythroid 2 related factor 2
ROS: reactive oxygen species
UPR: unfolded protein response

## References

[1] IARC (1990). IARC Monographs on the Valuation of the Carcinogenic Risks of Chemicals to Humans: Chromium, Nickel and Welding.

[2] Valko M, Rhodes CJ, Moncol J, Izakovic M, Mazur M (2006). Free radicals, metals and antioxidants in oxidative stress-induced cancer. Chem Biol Interact.

[3] Efremenko AY, Campbell JL, Dodd DE, Oller AR, Clewell HJ (2017). Time- and concentration-dependent genomic responses of the rat airway to inhaled nickel sulfate. Environ Mol Mutagen.

[4] Kasprzak KS, Sunderman FW, Salnikow K (2003). Nickel carcinogenesis. Mutat Res.

[5] Goodman JE, Prueitt RL, Dodge DG, Thakali S (2009). Carcinogenicity assessment of water-soluble nickel compounds. Crit Rev Toxicol.

[6] Salnikow K, Kasprzak KS (2007). Nickel-Dependent Gene Expression. Nickel and Its Surprising Impact in Nature.

[7] Salnikow K, Zhitkovich A (2008). Genetic and epigenetic mechanisms in metal carcinogenesis and cocarcinogenesis: nickel, arsenic, and chromium. Chem Res Toxicol.

[8] Costa M, Davidson TL, Chen H, Ke Q, Zhang P, Yan Y, Huang C, Kluz T (2005). Nickel carcinogenesis: epigenetics and hypoxia signaling. Mutat Res.

[9] Das K, Das S, Dhundasi S (2008). Nickel, its adverse health effects & oxidative stress. Indian J Med Res.

[10] Kim HL, Seo YR (2012). Molecular and genomic approach for understanding the gene-environment interaction between Nrf2 deficiency and carcinogenic nickel-induced DNA damage. Oncol Rep.

[11] Lewis JB, Messer RL, McCloud VV, Lockwood PE, Hsu SD, Wataha JC (2006). Ni(II) activates the Nrf2 signaling pathway in human monocytic cells. Biomaterials.

[12] Zhao X, Wen L, Dong M, Lu X (1653). Sulforaphane activates the cerebral vascular Nrf2-ARE pathway and suppresses inflammation to attenuate cerebral vasospasm in rat with subarachnoid hemorrhage. Brain Res.

[13] Menegon S, Columbano A, Giordano S (2016). The dual roles of NRF2 in Cancer. Trends Mol Med.

[14] Cho H-Y, Reddy SP, Kleeberger SR (2006). Nrf2 defends the lung from oxidative stress. Antioxid Redox Signal.

[15] Son YO, Pratheeshkumar P, Divya SP, Zhang Z, Shi X (2017). Nuclear factor erythroid 2-related factor 2 enhances carcinogenesis by suppressing apoptosis and promoting autophagy in nickel-transformed cells. J Biol Chem.

[16] Yu X, Robinson JF, Sidhu JS, Hong S, Faustman EM (2010). A system-based comparison of gene expression reveals alterations in oxidative stress, disruption of ubiquitin-proteasome system and altered cell cycle regulation after exposure to cadmium and Methylmercury in mouse embryonic fibroblast. Toxicol Sci.

[17] Jensen LJ, Kuhn M, Stark M, Chaffron S, Creevey C, Muller J, Doerks T, Julien P, Roth A, Simonovic M (2009). STRING 8--a global view on proteins and their functional interactions in 630 organisms. Nucleic Acids Res.

[18] Snel B, Lehmann G, Bork P, Huynen MA (2000). STRING: a web-server to retrieve and display the repeatedly occurring neighbourhood of a gene. Nucleic Acids Res.

[19] Feltes BC, JdF P, Notari DL, Bonatto D (2013). Toxicological Effects of the Different Substances in Tobacco Smoke on Human Embryonic Development by a Systems Chemo-Biology Approach. PLOS ONE.

[20] Cempel M, Nikel G. Nickel: a review of its sources and environmental toxicology. Pol J Environ Stud. 2006;15.

[21] Group IW: Nickel and nickel compounds. In IARC Monographs on the Evaluation of Carcinogenic Risks to Humans, No 100C*.* Edited by humans. IWGotEoCRt. Lyon (FR): International Agency for Research on Cancer; 2012.

[22] Schaumlöffel D (2012). Nickel species: analysis and toxic effects. J Trace Elem Med Biol.

[23] Stark C, Breitkreutz B-J, Reguly T, Boucher L, Breitkreutz A, Tyers M (2006). BioGRID: a general repository for interaction datasets. Nucleic Acids Res.

[24] Shannon P, Markiel A, Ozier O, Baliga NS, Wang JT, Ramage D, Amin N, Schwikowski B, Ideker T (2003). Cytoscape: a software environment for integrated models of biomolecular interaction networks. Genome Res.

[25] Bader GD, Hogue CW (2003). An automated method for finding molecular complexes in large protein interaction networks. BMC Bioinformatics.

[26] Cline MS, Smoot M, Cerami E, Kuchinsky A, Landys N, Workman C, Christmas R, Avila-Campilo I, Creech M, Gross B (2007). Integration of biological networks and gene expression data using Cytoscape. Nat Protoc.

[27] Rosado JO, Henriques JAP, Bonatto D (2011). A systems pharmacology analysis of major chemotherapy combination regimens used in gastric cancer treatment: predicting potential new protein targets and drugs. Curr Cancer Drug Targets.

[28] Scardoni G, Petterlini M, Laudanna C (2009). Analyzing biological network parameters with CentiScaPe. Bioinformatics (Oxford, England).

[29] Barthelemy M (2004). Betweenness centrality in large complex networks. The European physical journal B.

[30] Rubinov M, Sporns O (2010). Complex network measures of brain connectivity: uses and interpretations. Neuroimage.

[31] Kahl VFS, da Silva J, da Silva FR (2016). Influence of exposure to pesticides on telomere length in tobacco farmers: a biology system approach. Mutation Research/Fundamental and Molecular Mechanisms of Mutagenesis.

[32] Ashburner M, Ball CA, Blake JA, Botstein D, Butler H, Cherry JM, Davis AP, Dolinski K, Dwight SS, Eppig JT (2000). Gene ontology: tool for the unification of biology. The gene ontology consortium. Nat Genet.

[33] Maere S, Heymans K, Kuiper M (2005). BiNGO: a Cytoscape plugin to assess overrepresentation of gene ontology categories in biological networks. Bioinformatics (Oxford, England).

[34] Benjamini Yoav, Hochberg Yosef (1995). Controlling the False Discovery Rate: A Practical and Powerful Approach to Multiple Testing. Journal of the Royal Statistical Society: Series B (Methodological).

[35] Evans RM, Davies PJ, Costa M (1982). Video time-lapse microscopy of phagocytosis and intracellular fate of crystalline nickel sulfide particles in cultured mammalian cells. Cancer Res.

[36] Kasprzak K, Sunderman JF (1977). Mechanisms of dissolution of nickel subsulfide in rat serum. Res Commun Chem Pathol Pharmacol.

[37] Goodman JE, Prueitt RL, Thakali S, Oller AR (2011). The nickel ion bioavailability model of the carcinogenic potential of nickel-containing substances in the lung. Crit Rev Toxicol.

[38] Costa M, Mollenhauer HH (1980). Carcinogenic activity of particulate nickel compounds is proportional to their cellular uptake. Science.

[39] Cangul H, Broday L, Salnikow K, Sutherland J, Peng W, Zhang Q, Poltaratsky V, Yee H, Zoroddu MA, Costa M (2002). Molecular mechanisms of nickel carcinogenesis. Toxicol Lett.

[40] Grisham M, Jourd'Heuil D, Wink D (2000). chronic inflammation and reactive oxygen and nitrogen metabolism–implications in DNA damage and mutagenesis. Aliment Pharmacol Ther.

[41] Huang X, Klein CB, Costa M (1994). Crystalline Ni3S2 specifically enhances the formation of oxidants in the nuclei of CHO cells as detected by dichlorofluorescein. Carcinogenesis.

[42] Costa M, Salnikow K, Sutherland JE, Broday L, Peng W, Zhang Q, Kluz T: The role of oxidative stress in nickel and chromate genotoxicity**.** In Oxygen/Nitrogen Radicals*:* Cell Injury and Disease*.* Springer; 2002: 265–275. 12162442

[43] Cavallo D, Ursini CL, Setini A, Chianese C, Piegari P, Perniconi B, Iavicoli S (2003). Evaluation of oxidative damage and inhibition of DNA repair in an in vitro study of nickel exposure. Toxicol in Vitro.

[44] Saini S, Nair N, Saini MR (2014). Prenatal exposure to nickel on pregnant Swiss albino mice and fetal development. Toxicol Environ Chem.

[45] Saini S, Nair N, Saini MR (2013). Embryotoxic and teratogenic effects of nickel in Swiss albino mice during organogenetic period. Biomed Res Int.

[46] Kim K, Wang CH, Ok YS, Lee SE (2019). Heart developmental toxicity by carbon black waste generated from oil refinery on zebrafish embryos (Danio rerio): combined toxicity on heart function by nickel and vanadium. J Hazard Mater.

[47] Mussotter F, Tomm JM, El Ali Z, Pallardy M, Kerdine-Römer S, Götz M, von Bergen M, Haase A, Luch A (2016). Proteomics analysis of dendritic cell activation by contact allergens reveals possible biomarkers regulated by Nrf2. Toxicol Appl Pharmacol.

[48] Lin C-YCT-H (1998). NICKEL TOXICITY TO HUMAN TERM PLACENTA: IN VITRO STUDY ON LIPID PEROXIDATION. J Toxic Environ Health A.

[49] Liu C-M, Zheng G-H, Ming Q-L, Chao C, Sun J-M (2013). Sesamin protects mouse liver against nickel-induced oxidative DNA damage and apoptosis by the PI3K-Akt pathway. J Agric Food Chem.

[50] Kweider N, Huppertz B, Rath W, Lambertz J, Caspers R, ElMoursi M, Pecks U, Kadyrov M, Fragoulis A, Pufe T (2017). The effects of Nrf2 deletion on placental morphology and exchange capacity in the mouse. J Matern Fetal Neonatal Med.

[51] Sarkar B. Heavy metals in the environment: Taylor & Francis; 2002.

[52] Sutherland JE, Peng W, Zhang Q, Costa M (2001). The histone deacetylase inhibitor trichostatin a reduces nickel-induced gene silencing in yeast and mammalian cells. Mutat Res.

[53] Hattori N, Ushijima T (2014). Compendium of aberrant DNA methylation and histone modifications in cancer. Biochem Biophys Res Commun.

[54] Ke Q, Ellen TP, Costa M (2008). Nickel compounds induce histone ubiquitination by inhibiting histone deubiquitinating enzyme activity. Toxicol Appl Pharmacol.

[55] Ke Q, Davidson T, Chen H, Kluz T, Costa M (2006). Alterations of histone modifications and transgene silencing by nickel chloride. Carcinogenesis.

[56] Ma L, Bai Y, Pu H, Gou F, Dai M, Wang H, He J, Zheng T, Cheng N (2015). Histone methylation in nickel-smelting industrial workers. PLoS One.

[57] Kang J, Zhang Y, Chen J, Chen H, Lin C, Wang Q, Ou Y (2003). Nickel-induced histone hypoacetylation: the role of reactive oxygen species. Toxicol Sci.

[58] Mercado N, Thimmulappa R, Thomas CMR, Fenwick PS, Chana KK, Donnelly LE, Biswal S, Ito K, Barnes PJ (2011). Decreased histone deacetylase 2 impairs Nrf2 activation by oxidative stress. Biochem Biophys Res Commun.

[59] Wang B, Zhu X, Kim Y, Li J, Huang S, Saleem S, Li R-c, Xu Y, Dore S, Cao W (2012). Histone deacetylase inhibition activates transcription factor Nrf2 and protects against cerebral ischemic damage. Free Radic Biol Med.

[60] Vomund S, Schäfer A, Parnham MJ, Brüne B, von Knethen A (2017). Nrf2, the master regulator of anti-oxidative responses. Int J Mol Sci.

[61] Khobta A, Epe B (2012). Interactions between DNA damage, repair, and transcription. Mutat Res.

[62] House NCM, Koch MR, Freudenreich CH (2014). Chromatin modifications and DNA repair: beyond double-strand breaks. Front Genet.

[63] Linder T (1999). Direct Ni2+ antigen formation on cultured human dendritic cells. Immunology.

[64] Medici S, Peana M, Nurchi VM, Zoroddu MA (2013). The involvement of amino acid side chains in shielding the nickel coordination site: an NMR study. Molecules.

[65] Thierse H-J, Gamerdinger K, Junkes C, Guerreiro N, Weltzien HU (2005). T cell receptor (TCR) interaction with haptens: metal ions as non-classical haptens. Toxicology.

[66] Goebeler M, Meinardus-Hager G, Roth J, Goerdt S, Sorg C (1993). Nickel chloride and cobalt chloride, two common contact sensitizers, directly induce expression of intercellular adhesion molecule-1 (ICAM-1), vascular cell adhesion molecule-1 (VCAM-1), and endothelial leukocyte adhesion molecule (ELAM-1) by endothelial cells. J Investig Dermatol.

[67] Chana M, Lewis JB, Davis R, Elam Y, Hobbs D, Lockwood PE, Wataha JC, Messer RL (2018). Biological effects of Ni(II) on monocytes and macrophages in normal and hyperglycemic environments. J Biomed Mater Res A.

[68] Cruz MT, Goncalo M, Figueiredo A, Carvalho AP, Duarte CB, Lopes MC (2004). Contact sensitizer nickel sulfate activates the transcription factors NF-kB and AP-1 and increases the expression of nitric oxide synthase in a skin dendritic cell line. Exp Dermatol.

[69] Huang Y, Davidson G, Li J, Yan Y, Chen F, Costa M, Chen LC, Huang C (2002). Activation of nuclear factor-kappaB and not activator protein-1 in cellular response to nickel compounds. Environ Health Perspect.

[70] Lewis JB, Wataha JC, McCloud V, Lockwood PE, Messer RL, Tseng WY (2005). Au(III), Pd(II), Ni(II), and hg(II) alter NF kappa B signaling in THP1 monocytic cells. J Biomed Mater Res A.

[71] Lewis JB, Messer RL, Pitts L, Hsu SD, Hansen JM, Wataha JC: Ni (II) ions dysregulate cytokine secretion from human monocytes**.** Journal of Biomedical Materials Research Part B: Applied Biomaterials: An Official Journal of The Society for Biomaterials, The Japanese Society for Biomaterials, and The Australian Society for Biomaterials and the Korean Society for Biomaterials 2009, 88**:**358–365. 10.1002/jbm.b.31063 18437699

[72] Pan H, Wang H, Wang X, Zhu L, Mao L (2012). The absence of Nrf2 enhances NF-kappaB-dependent inflammation following scratch injury in mouse primary cultured astrocytes. Mediat Inflamm.

[73] Jin W, Wang H, Yan W, Xu L, Wang X, Zhao X, Yang X, Chen G, Ji Y (2008). Disruption of Nrf2 enhances upregulation of nuclear factor-kappaB activity, proinflammatory cytokines, and intercellular adhesion molecule-1 in the brain after traumatic brain injury. Mediat Inflamm.

[74] Yu M, Li H, Liu Q, Liu F, Tang L, Li C, Yuan Y, Zhan Y, Xu W, Li W (2011). Nuclear factor p65 interacts with Keap1 to repress the Nrf2-ARE pathway. Cell Signal.

[75] Oller AR, Costa M, Oberdörster G (1997). Carcinogenicity assessment of selected nickel compounds. Toxicol Appl Pharmacol.

[76] Grabbe S, Schwarz T (1998). Immunoregulatory mechanisms involved in elicitation of allergic contact hypersensitivity. Immunol Today.

[77] Efremenko A, Campbell J, Dodd D, Oller A, Clewell H (2014). Time-and concentration-dependent genomic responses of the rat airway to inhaled nickel subsulfide. Toxicol Appl Pharmacol.

[78] Cao SS, Kaufman RJ (2014). Endoplasmic reticulum stress and oxidative stress in cell fate decision and human disease. Antioxid Redox Signal.

[79] Xu C, Bailly-Maitre B, Reed JC (2005). Endoplasmic reticulum stress: cell life and death decisions. J Clin Investig.

[80] Simard J-C, Vallieres F, De Liz R, Lavastre V, Girard D (2015). Silver nanoparticles induce degradation of the endoplasmic reticulum stress sensor activating transcription factor-6 leading to activation of the NLRP-3 inflammasome. J Biol Chem.

[81] Lu T-H, Tseng T-J, Su C-C, Tang F-C, Yen C-C, Liu Y-Y, Yang C-Y, Wu C-C, Chen K-L, Hung D-Z (2014). Arsenic induces reactive oxygen species-caused neuronal cell apoptosis through JNK/ERK-mediated mitochondria-dependent and GRP 78/CHOP-regulated pathways. Toxicol Lett.

[82] Xu B, Shan M, Wang F, Deng Y, Liu W, Feng S, Yang T-Y, Xu Z-F (2013). Endoplasmic reticulum stress signaling involvement in manganese-induced nerve cell damage in organotypic brain slice cultures. Toxicol Lett.

[83] Le Quynh Giang, Ishiwata-Kimata Yuki, Kohno Kenji, Kimata Yukio (2016). Cadmium impairs protein folding in the endoplasmic reticulum and induces the unfolded protein response. FEMS Yeast Research.

[84] Hiramatsu N, Kasai A, Du S, Takeda M, Hayakawa K, Okamura M, Yao J, Kitamura M (2007). Rapid, transient induction of ER stress in the liver and kidney after acute exposure to heavy metal: evidence from transgenic sensor mice. FEBS Lett.

[85] Guo H, Cui H, Peng X, Fang J, Zuo Z, Deng J, Wang X, Wu B, Chen K, Deng J (2016). Nickel chloride (NiCl2) induces endoplasmic reticulum (ER) stress by activating UPR pathways in the kidney of broiler chickens. Oncotarget.

[86] Tamás MJ, Sharma SK, Ibstedt S, Jacobson T, Christen P (2014). Heavy metals and metalloids as a cause for protein misfolding and aggregation. Biomolecules.

[87] Ke Q, Davidson T, Kluz T, Oller A, Costa M (2007). Fluorescent tracking of nickel ions in human cultured cells. Toxicol Appl Pharmacol.

[88] Kawanishi S, Oikawa S, Inoue S, Nishino K (2002). Distinct mechanisms of oxidative DNA damage induced by carcinogenic nickel subsulfide and nickel oxides. Environ Health Perspect.

[89] Zhang K, Kaufman RJ (2006). The unfolded protein response: a stress signaling pathway critical for health and disease. Neurology.

[90] Chang C-W, Chen Y-S, Tsay Y-G, Han C-L, Chen Y-J, Yang C-C, Hung K-F, Lin C-H, Huang T-Y, Kao S-Y (2018). ROS-independent ER stress-mediated NRF2 activation promotes Warburg effect to maintain stemness-associated properties of cancer-initiating cells. Cell Death Dis.

[91] Mota SI, Costa RO, Ferreira IL, Santana I, Caldeira GL, Padovano C, Fonseca AC, Baldeiras I, Cunha C, Letra L (1852). Oxidative stress involving changes in Nrf2 and ER stress in early stages of Alzheimer's disease. Biochim Biophys Acta.

[92] Angel P, Karin M (1991). The role of Jun, Fos and the AP-1 complex in cell-proliferation and transformation. Biochim Biophys Acta.

[93] Hai T, Curran T (1991). Cross-family dimerization of transcription factors Fos/Jun and ATF/CREB alters DNA binding specificity. Proc Natl Acad Sci.

[94] Barchowsky A, O'Hara KA (2003). Metal-induced cell signaling and gene activation in lung diseases. Free Radic Biol Med.

[95] Salnikow K, Davidson T, Zhang Q, Chen LC, Su W, Costa M (2003). The involvement of hypoxia-inducible transcription Factor-1-dependent pathway in nickel carcinogenesis. Cancer Res.

[96] Ding J, Zhang X, Li J, Song L, Ouyang W, Zhang D, Xue C, Costa M, Meléndez JA, Huang C (2006). Nickel compounds render anti-apoptotic effect to human bronchial epithelial Beas-2B cells by induction of cyclooxygenase-2 through an IKKβ/p65-dependent and IKKα-and p50-independent pathway. J Biol Chem.

[97] Sigel A, Sigel H, Sigel RKO: Nickel and Its Surprising Impact in Nature*.* Wiley; 2007.

[98] Aiba S, Manome H, Nakagawa S, Mollah ZU, Mizuashi M, Ohtani T, Yoshino Y, Tagami H (2003). p38 mitogen-activated protein kinase and extracellular signal-regulated kinases play distinct roles in the activation of dendritic cells by two representative haptens, NiCl2 and 2,4-dinitrochlorobenzene. J Invest Dermatol.

[99] Grösch S, Kaina B (1999). Transcriptional activation of apurinic/apyrimidinic endonuclease (ape, Ref-1) by oxidative stress requires CREB. Biochem Biophys Res Commun.

[100] Mayr B, Montminy M (2001). Transcriptional regulation by the phosphorylation-dependent factor CREB. Nat Rev Mol Cell Biol.

[101] Kikuchi D, Tanimoto K, Nakayama K (2016). CREB is activated by ER stress and modulates the unfolded protein response by regulating the expression of IRE1α and PERK. Biochem Biophys Res Commun.

[102] Ge Y, Bruno M, Haykal-Coates N, Wallace K, Andrews D, Swank A, Winnik W, Ross JA (2016). Proteomic assessment of biochemical pathways that are critical to nickel-induced toxicity responses in human epithelial cells. PLoS One.

[103] Wang J, Maldonado MA (2006). The ubiquitin-proteasome system and its role in inflammatory and autoimmune diseases. Cell Mol Immunol.

[104] Reinstein E (2004). Immunologic aspects of protein degradation by the ubiquitin-proteasome system. Isr Med Assoc J.

[105] Menéndez-Benito V, Verhoef LGGC, Masucci MG, Dantuma NP (2005). Endoplasmic reticulum stress compromises the ubiquitin–proteasome system. Hum Mol Genet.

[106] Hart LS, Cunningham JT, Datta T, Dey S, Tameire F, Lehman SL, Qiu B, Zhang H, Cerniglia G, Bi M (2012). ER stress–mediated autophagy promotes Myc-dependent transformation and tumor growth. J Clin Invest.

[107] Gao P, Tchernyshyov I, Chang T-C, Lee Y-S, Kita K, Ochi T, Zeller KI, De Marzo AM, Van Eyk JE, JTJN M (2009). c-Myc suppression of miR-23a/b enhances mitochondrial glutaminase expression and glutamine metabolism. Nature.

[108] Meyer Natalie, Penn Linda Z. (2008). Reflecting on 25 years with MYC. Nature Reviews Cancer.

[109] Iritani BM, Eisenman RNJPotNAoS: c-Myc enhances protein synthesis and cell size during B lymphocyte development. 1999, 96:13180–13185. 10.1073/pnas.96.23.13180 PMC23921 10557294

[110] Chan J. C., Hannan K. M., Riddell K., Ng P. Y., Peck A., Lee R. S., Hung S., Astle M. V., Bywater M., Wall M., Poortinga G., Jastrzebski K., Sheppard K. E., Hemmings B. A., Hall M. N., Johnstone R. W., McArthur G. A., Hannan R. D., Pearson R. B. (2011). AKT Promotes rRNA Synthesis and Cooperates with c-MYC to Stimulate Ribosome Biogenesis in Cancer. Science Signaling.

[111] Zhu X, Huang L, Gong J, Shi C, Wang Z, Ye B, Xuan A, He X, Long D, Zhu X (2017). NF-κB pathway link with ER stress-induced autophagy and apoptosis in cervical tumor cells. Cell Death Discovery.

[112] Liu G-H, Qu J, Shen X (2008). NF-κB/p65 antagonizes Nrf2-ARE pathway by depriving CBP from Nrf2 and facilitating recruitment of HDAC3 to MafK. Biochimica et Biophysica Acta (BBA) - Molecular Cell Research.

